# Psychophysiological responses to treadwall and indoor wall climbing in adult female climbers

**DOI:** 10.1038/s41598-021-82184-6

**Published:** 2021-01-29

**Authors:** Jiří Baláš, Jan Gajdošík, Dominika Krupková, Leona Chrastinová, Alžběta Hlaváčková, Radka Bačáková, David Giles

**Affiliations:** 1 Faculty of Physical Education and Sport, Charles University in Prague, José Martího 31, 16252 Prague 6, Czech Republic; 2 Institute of Hematology and Blood Transfusion, Prague, Czech Republic; 3 Lattice Training Ltd, Chesterfield, Derbyshire UK

**Keywords:** Cardiovascular biology, Respiration, Psychology

## Abstract

The purpose of the study was to compare the psychophysiological response of climbers of a range of abilities (lower grade to advanced) when ascending identical climbing routes on a climbing wall and a rotating treadwall. Twenty-two female climbers (31.2 ± 9.4 years; 60.5 ± 6.5 kg; 168.6 ± 5.7 cm) completed two identical 18 m climbing trials (graded 4 on the French Sport scale) separated by 1 week, one on the treadwall (climbing low to the ground) and the other on the indoor wall (climbing in height). Indirect calorimetry, venous blood samples and video-analysis were used to assess energy cost, hormonal response and time-load characteristics. Energy costs were higher during indoor wall climbing comparing to those on the treadwall by 16% (*P* < 0.001, $\upmu _{{\text{p}}}^{2}$ = 0.48). No interaction of climbing ability and climbing condition were found. However, there was an interaction for climbing ability and post-climbing catecholamine concentration (*P* < 0.01, $\upmu _{{\text{p}}}^{2}$ = 0.28). Advanced climbers’ catecholamine response increased by 238% and 166% with respect to pre-climb values on the treadwall and indoor wall, respectively; while lower grade climbers pre-climb concentrations were elevated by 281% and 376% on the treadwall and indoor wall, respectively. The video analysis showed no differences in any time-motion variables between treadwall and indoor wall climbing. The study demonstrated a greater metabolic response for indoor wall climbing, however, the exact mechanisms are not yet fully understood.

## Introduction

Performance in climbing is not only dictated by the physical demands of an ascent, but also by psychological, technical and tactical factors^1,2^. The complexity of the concomitant physiological and psychological response occurring when climbers attempt to ascend routes complicates the assessment of factors occurring purely as a result of the physical load^3–8^. Consequently, there is a need for research exploring the interaction between physiological and psychological (psychophysiological) responses in climbing and climbing analogous tasks.

The scarcity of research exploring the psychophysiological response of climbers, in comparison to that which explores the physical characteristics, presents a challenge for both coaches and researchers. While differences in the psychophysiological response of varying ability groups of climbers when manipulating the means of protecting a climber in the event of a fall (style of ascent) have been described in the literature^3,8^, these studies have made a number of assumptions about the nature of the climbing task involved. For instance, they do not take into consideration other psychological factors that may influence performance, such as the ability to execute physical/technical climbing movements while high above the ground on a route. To date, no comparison to an analogous task that requires the same physical demand, without the need to ascend a route have been made—such a task would allow for the isolation of the climber’s response to ascents of climbing walls that occur due to only the task’s physical demands from the physiological, technical tactical demands required of them. A treadwall may present such an opportunity.

In other areas of climbing research, treadwalls (continuous climbing walls, comparable to a vertical treadmill) have been used to assess factors including the cardiorespiratory and haemodynamic response to climbing to exhaustion^9–12^. Treadwalls are rotating climbing walls that allow for continuous climbing on a vertical belt. A mechanical treadwall has many advantages over an ascent of a wall in an indoor gym as it enables control for climbing speed and for belt inclination. Climbing is undertaken low to the ground and requires only limited equipment such as shoes and a chalk bag. However, to date, it has been assumed that treadwall climbing induces similar physiological response as climbing on the wall and previous research has assumed that the treadwall climbing has a direct transfer to actual indoor wall climbing^10,12^. Coaches and researchers should always consider whether physiological responses result from physical effort alone or from a combination of both psychological and physiological factors. This is particularly relevant when prescribing exercise intensity in fitness-focused programs, as apparently high intensity climbing (high physiological response resulting from psychological stress) may induce trivial muscle or cardiovascular adaptations.

Therefore, the purpose of the present study was to compare the psychophysiological response of climbers of a range of abilities (lower grade to advanced) when ascending identical climbing routes on an 18 m climbing wall and a rotating 3.2 m high treadwall. Previous research has demonstrated that the style of ascent and fall potential increase stress hormone response, heart rate (HR) and oxygen consumption ($\dot{V}$O_2_) in lower grade climbers, but not in advanced and elite climbers^5,7,8,13,14^. Based on these previous findings, we hypothesized that lower grade climbers will show greater psychophysiological response in top rope (TR) indoor route ascent than on treadwall due to stress from height, although, more advanced climbers will show no differences.

## Methods

### Participants

Twenty-two female climbers volunteered to participate in the study. Climbers self-reported their on-sight climbing ability from 7 to 21 on IRCRA scale (International Rock Climbing Research Association); 4–7c on French sport grades; and were divided by median (13 IRCRA) into lower grade + intermediate (N = 11) and intermediate + advanced climbers (N = 11)^15^. To simplify, the groups are referred to only as lower grade or advanced throughout the text. Lower grade climbers characteristics (age: 32.6 ± 11.3 years; body mass 63.0 ± 5.7 kg; height 168.9 ± 5.7 cm) were similar to those of the advanced group (age: 29.8 ± 7.4 years; body mass 58.1 ± 6.5 kg; height 168.3 ± 5.9 cm) and no statistical differences were stated. Participants were asked to refrain from all climbing and intense physical activities for 24 h prior to testing; they were instructed to maintain the same diet for the preceding two days and to abstain from any ergogenic aid such as caffeine on the day of testing. To account for potential influences of hormonal status on study outcomes, pre-menopausal women were studied during the early follicular phase of their menstrual cycle or during the placebo phase of oral contraceptive use.

Sample size calculations using G∗Power (version 3.1.9.7) were based on total catecholamine concentration. With power (1 − β error) set at 0.95, α-level set at 0.05 and a previously reported^5^ effect size of 1.3 (Cohen’s *d*) in catecholamine changes between two differing climbing task, a sample of 12 was required. However, due to expected less pronounced changes in catecholamine concentration, and the potential for missing data, we recruited 22 participants.

The study conformed to the recommendations of the local Ethics Committee in accordance with the Declaration of Helsinki. All participants were informed of the experimental risks and provided written informed consent prior to the commencement of data collection.

### Experimental design

**Figure 1:**
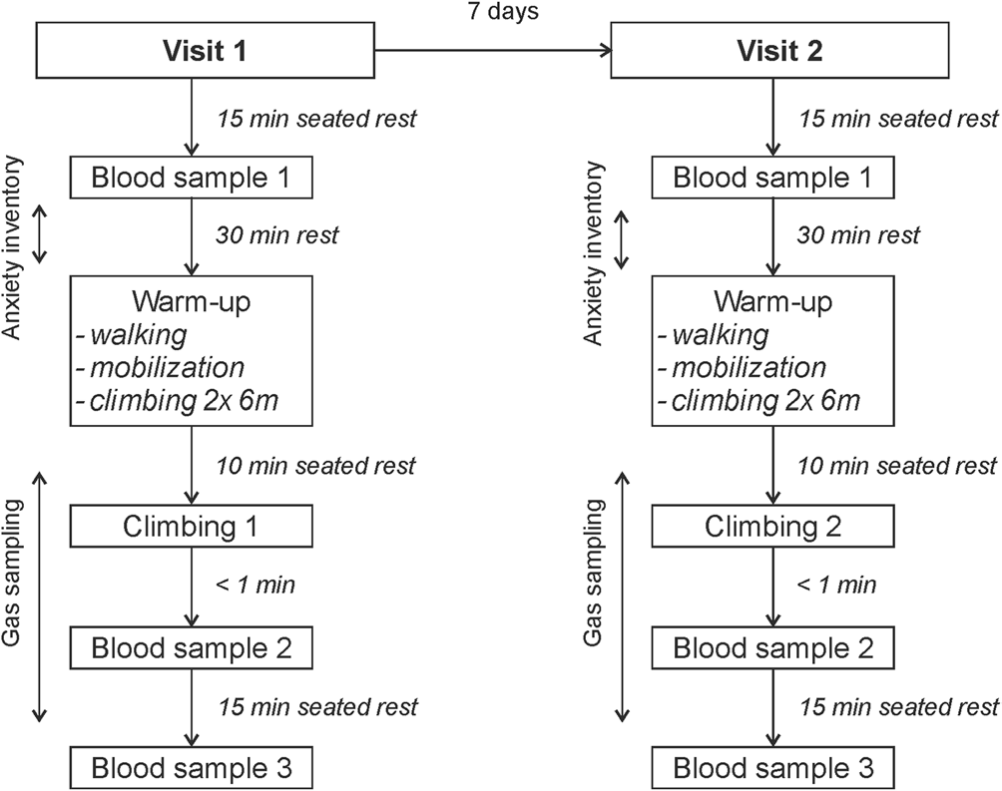
Experimental plan of the study with randomised climbing conditions—indoor wall or treadwall.

Participants visited the climbing gym, where the study was taking place, on two occasions separated by 7 days in a randomised counterbalanced crossover design. Both visits followed the same time schedule as depicted at Fig. 1 and all testing was undertaken at the same time of day ± 30 min. On the first visit, the purpose of the study was explained to participants, any questions that they may have had answered, and when clear of all facts they signed informed consent. After 15 min of seated rest, the initial venous blood sample was collected, this was followed by another 30 min of seated rest during which time participants completed the anxiety inventories. Following the period of rest participants completed a standardised warm-up (brisk 5 min walk, 5 min of mobilisation exercises, 2 trials of 6 m on the climbing route at self-selected and given speed). Afterwards, climbers were randomly assigned to ascend an 18 m long route either on the wall or on the treadwall. Immediately after and 15 min after the climb, the second and third venous blood samples were collected, respectively. Based on authors’ practical experience, it was assumed that climbing at 18 m vertical wall will elicit mental stress response particularly in lower grade climbers not habituated to height exposure and may even increase pre-climb anxiety levels. Therefore, intra-individual responses during both climbing conditions may help to explain the role of psychological factors, as the physical load was identical. Moreover, inter-individual responses may reflect both physical and mental adaptations to climbing training.

### Climbing routes

The routes attempted by the participants on the indoor wall and treadwall were identical and were climbed at a set speed of 4 m min^−1^. The routes were both vertical, 18 m in length and the difficulty was estimated at 7 on the IRCRA scale (5.4 on Yosemite Decimal Scale; 4 on the Sport grade), so the total climbing time was 4.5 min. The starting hold was at 1.5 m off the ground for both conditions, a licensed route setter set three identical technically simple sequences 6 m in length with vertical positive holds in order to reduce any learning effect^16^. The route on the indoor wall was protected using a TR and participants belayed by an experienced instructor. Colour marks were attached every meter to the wall to control visually the speed of ascent (1 m per 15 s). An instructor provided the climber audible feedback throughout their ascent to assist with maintaining this pace. The climber finished the route with both hands at a height of 19.5 m (19.5 − 1.5 = 18 m). The treadwall (ClimbStation, Finland) is a motorized climbing ergometer 3.2 m high with 6 m long plate belt enabling control of the inclination and speed of locomotion. At their highest point while climbing on the treadwall, climbers were a maximum of 0.5 m over the security mattress. When an accidental slip from a hold occurred, the climber was asked to immediately continue the climb (this only occurred for one participant once during the experiment). Climbers were allowed to use chalk in both conditions.

### Gas analysis and energy cost

Expired minute ventilation ($\dot{V}$_E_), $\dot{V}$O_2_, and carbon dioxide production ($\dot{V}$CO_2_) were measured by a portable breath-by-breath indirect calorimetry system (MetaMax 3B, Cortex Biophysic, Germany). The MetaMax 3B was attached onto the chest by a harness. Before each test, gas and volume calibration was performed according to manufacturer’s guidelines. The volume calibration was performed using a known 3L syringe and gas calibration was performed with a known gas mixture of 15% O_2_ and 5% CO_2_^17^. Data was collected continuously from 5 min pre-climb rest, climbing and 10 min seated recovery period. Averaged data over 20 s intervals was exported to Excel for further analysis. The mean from the last minute of the treadwall and gym climbing was used to represent $\dot{V}$O_2_ “steady state” of the activity. Excess post-exercise oxygen uptake (EPOC) was calculated from 10 min sitting rest as total recovery $\dot{V}$O_2_ lowered from resting $\dot{V}$O_2_. The resting $\dot{V}$O_2_ represented the lowest 1-min $\dot{V}$O_2_ from pre-climb rest or post-climb recovery. If the resting $\dot{V}$O_2_ differed in the two visit conditions, the lower value was used. Resting $\dot{V}$O_2_ was then subtracted from total climbing $\dot{V}$O_2_ to have net climbing $\dot{V}$O_2_. The net climbing energy cost (EC) was subsequently computed from net climbing $\dot{V}$O_2_ and EPOC using energy equivalent for oxygen of 4.924 kcal. RER was computed by dividing $\dot{V}$CO_2_ by $\dot{V}$O_2_. HR was monitored by the MetaMax 3B using a Polar heart transmitter belt (Polar Electro OY, Finland).

### Hormonal response

Venous blood (2.7 ml) was drawn by venipuncture into polypropylene tubes coated with ethylenediaminetetraacetic acid. Plasma was obtained by the centrifugation of blood samples (15 min, 3000×*g*, 20 °C). Plasma was frozen at − 80 °C and was only thawed prior to assays. From the plasma concentrations of adrenaline (epinephrine), noradrenaline (norepinephrine), dopamine, and cortisol were determined. Cortisol and catecholamines were analysed by liquid chromatography (LC) and mass spectrometry (MS). LC was performed using LC system Shimadzu Prominence (Shimadzu, Czech Republic) equipped by column Atlantis dC18 [100 × 2.1 mm, 5 μm (Waters, Czech Republic)]. The detection system (MS detector QTRAP 4000; Sciex, Czech Republic) worked in the positive ionisation mode using selected reaction monitoring for compounds detection. Ion source temperature was 450 °C for cortisol and 600 °C for catecholamines, the ion source voltage was set at 5500 V for cortisol and 5000 V for catecholamines^5^. The analyses were completed by one researcher with following intra-assay and inter-assay coefficient of variation (intra CV and inter CV, respectively): cortisol intra CV = 4.7%, inter CV = 6.4%; noradrenaline intra CV = 5.9%, inter CV = 7.3%; adrenaline intra CV = 3.6%, inter CV = 6.6%; dopamine intra CV = 8.9%, inter CV = 9.7%.

### Time-motion analysis

For both conditions, a video recording (Canon Legria FS306, Ohta-ku Tokio, Japan; 25 frames per second) was made and subsequently hand contact with the hold was evaluated using video-analytic software (Dartfish HQ, Fribourg, Switzerland). Then, one-hand (left and right) and both-hand position time on the hold and the work/relief ratio for forearm muscles were calculated^18^.

### Anxiety and self-confidence assessment

The CSAI-2R, as revised by Cox et al.^19^ and TFAI as proposed by Cheng et al.^20^, were used to assess each individuals feelings of anxiety (somatic and cognitive), self-confidence and perceived control before the climb. Both inventories were completed by the climber after pre-climb blood sampling before the warm-up. CSAI-2R includes 17 items and TFAI 25 items which are scored on a Likert scale from 1 to 4 and from 1 to 5, respectively. The scores were combined to give a final score on 3 subscales: somatic anxiety, cognitive anxiety and self-confidence for CSAI-2R and cognitive anxiety (worry, self-focus), physiological anxiety (autonomous hyperactivity, somatic tension) and perceived control (regulatory dimension) for TFAI.

### Data analysis

Descriptive statistics (mean ± *s*) were used to assess basic anthropometric and training characteristics. Possible differences in these characteristics between lower grade and advanced climbers were assessed using a series of independent samples *t*-tests. The effect of climbing condition (indoor wall × treadwall) and climbing ability (lower grade x advanced) was evaluated based on mixed model (2 × 2) ANOVA with climbing condition as within-subject factor and ability level as between-subject factor. Further, pairwise comparisons were calculated by paired or independent samples t-test (LSD method). The relationship between metabolic and hormonal response variables was checked by way of Pearson correlation coefficients. Statistical significance was set at *P* ≤ 0.05. Cohen *d* and partial eta squared (µ_p_^2^) were used to assess size effect where value of 0.2, 0.5, > 0.8 represents small, moderate and large differences and 0.05, 0.10, > 0.20 represents small, intermediate and large effect, respectively.

### Ethics approval and consent to participate

The study conformed to the recommendations of the Ethics Committee (Charles University, Faculty of Physical Education and Sport) in accordance with the Declaration of Helsinki. All participants were informed of the experimental risks and provided written informed consent prior to the commencement of data collection.

## Results

**Figure 2:**
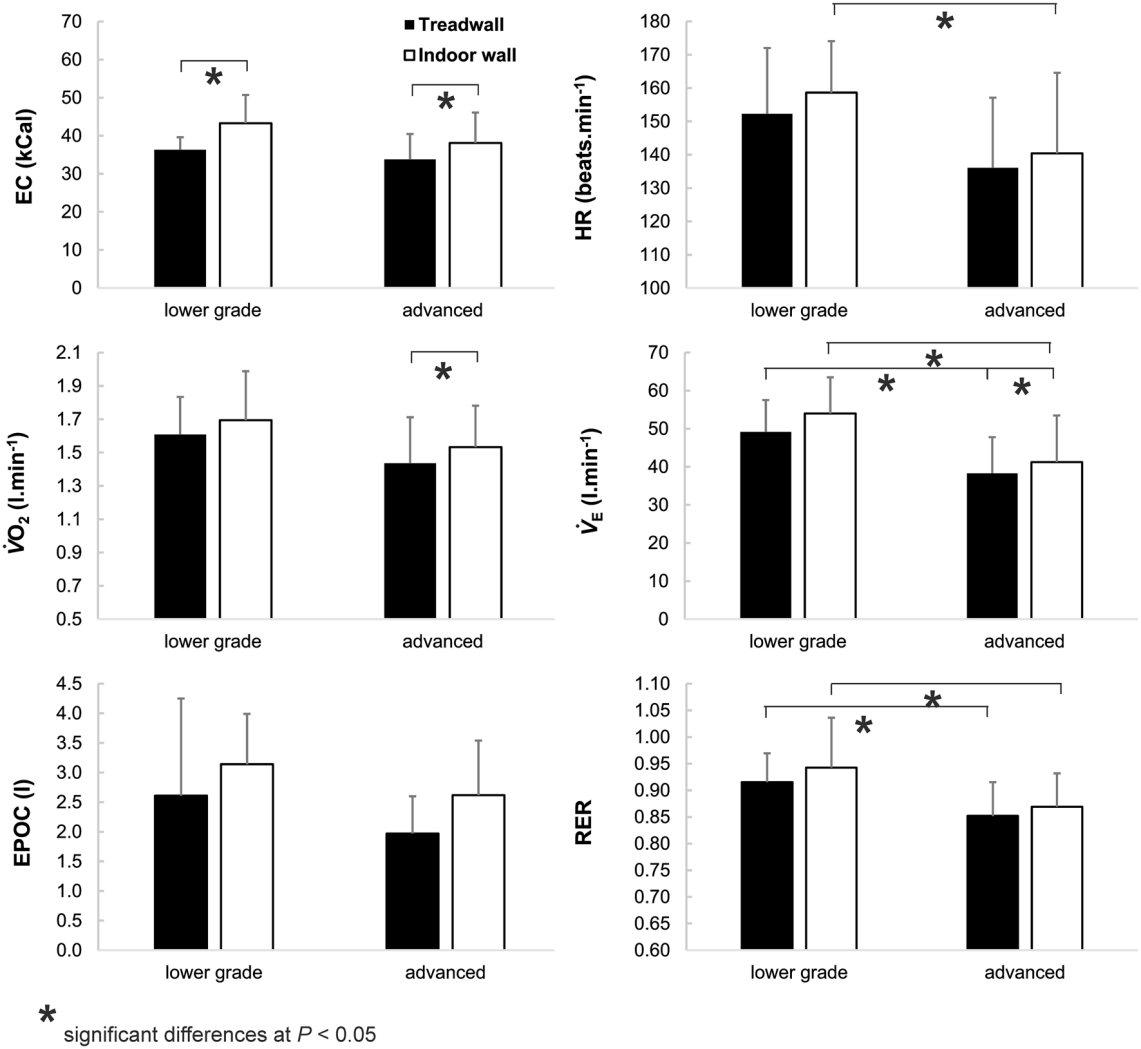
Mean (± *s*) oxygen consumption ($\dot{V}$O_2_), heart rate (HR), excess post-exercise oxygen consumption (EPOC), expired minute ventilation ($\dot{V}$_E_), energy cost (EC), and respiratory ratio (RER) during climbing on the treadwall and in climbing gym in lower grade and advanced climbers.

Advanced climbers practiced climbing significantly (*P* < 0.05) longer, spent more time training per week and preferred bouldering (Table 1). Participant’s physiological response was significantly greater during indoor wall climbing when compared to treadwall climbing for $\dot{V}$O_2_ (+ 6%; *P* = 0.03; $\upmu _{{\text{p}}}^{2}$ = 0.22), HR (+ 4%; *P* = 0.04; $\upmu _{{\text{p}}}^{2}$ = 0.20), $\dot{V}$_E_ (+ 9%; *P* = 0.01; $\upmu _{{\text{p}}}^{2}$ = 0.30), and EC (+ 16%; *P* < 0.001; $\upmu _{{\text{p}}}^{2}$ = 0.48). Furthermore, the lower grade climbers had significantly greater HR (*P* = 0.05; $\upmu _{{\text{p}}}^{2}$ = 0.18), $\dot{V}$_E_ (*P* = 0.01, $\upmu _{{\text{p}}}^{2}$ = 0.29), and RER (*P* = 0.02, $\upmu _{{\text{p}}}^{2}$ = 0.24) than advanced climbers. Pairwise comparisons (Fig. 2) showed statistically significant differences between treadwall and indoor wall climbing for more metabolic variables in the advance rather than the lower grade climbers. However, no interaction between climbing ability and climbing condition was found.

**Table 1:** Climbing ability and training characteristics (mean ± *s*) in lower grade and advanced climbers.

Climbers	Lower grade (N = 11)	Advanced (N = 11)	*P*	Cohen’s d
Ability (IRCRA Scale)	10 ± 2	16 ± 3	< 0.001	3.5
Experience (years)	6 ± 5	10 ± 7	0.176	0.8
Outdoor climbing (%)	27 ± 25	38 ± 20	0.295	0.6
Indoor climbing (%)	73 ± 25	62 ± 24	0.262	0.7
Sport climbing (%)	83 ± 26	56 ± 34	0.044	1.3
Bouldering (%)	17 ± 26	44 ± 34	0.044	1.3
Trainings (number per week)	1.5 ± 0.7	2.5 ± 1.2	0.021	1.5

*IRCRA* international rock climbing research association.

**Figure 3:**
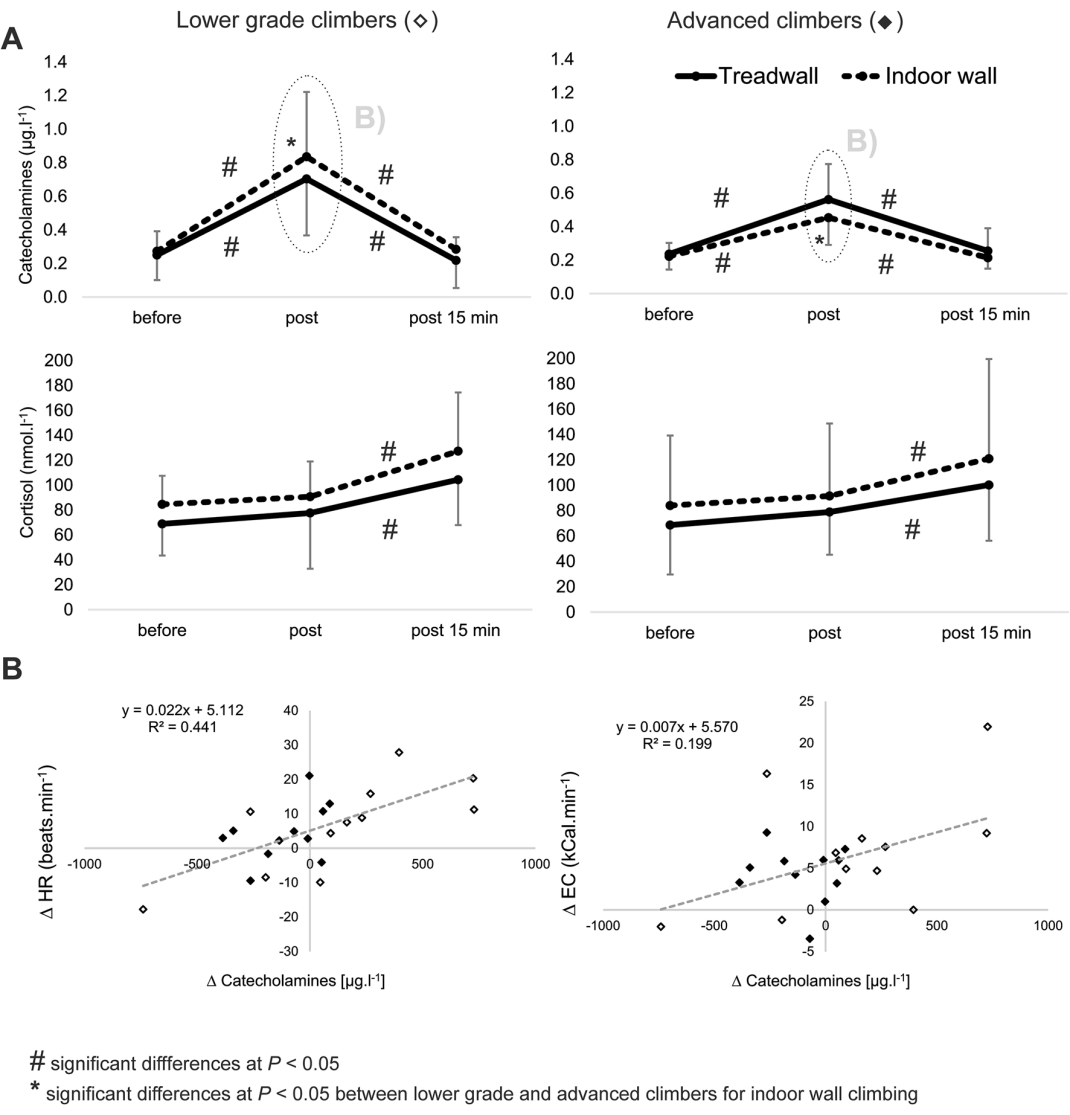
(**A**) Mean (± *s*) cortisol and catecholamine concentration before, immediately post and 15 min after climbing on the treadwall and in climbing gym in lower grade and advanced climbers; (**B**) Changes in heart rate (HR) and EC (energy cost) between treadwall (0 on the graph) and indoor wall climbing (full and empty quadrangles on the graph) in relationship with catecholamine changes. Empty and full quadrangles represent lower grade and advanced climbers, respectively.

Catecholamine concentration significantly increased immediately after both climbing conditions (*P* < 0.001, $\upmu _{{\text{p}}}^{2}$ = 0.81), while cortisol increased after 15 min recovery (*P* < 0.001, $\upmu _{{\text{p}}}^{2}$ = 0.68). There was an interaction for climbing ability and post-climbing catecholamine concentration (*P* < 0.01, $\upmu _{{\text{p}}}^{2}$ = 0.28), however, not for climbing condition. Advanced climber’s catecholamine response increased by 238% and 166% with respect to pre-climb concentrations on the treadwall and indoor wall, respectively; while lower grade climbers elevated the pre-climb concentration to 281% and 376% on the treadwall and indoor wall, respectively. Cortisol concentrations were elevated but not significantly throughout all measurements for the indoor wall condition than for the treadwall (Fig. 3).

An increased metabolic cost in response to the indoor wall condition was related to the changes in post climbing-catecholamine response and a significant relationship was found for HR and EC (Fig. 3). Climbers from the advanced ability group showed smaller variation in catecholamine and metabolic response between treadwall and indoor wall climbing, however, lower grade climbers were positioned at both extremes of the graph (Fig. 3). Some lower grade climbers demonstrated substantially increased catecholamine efflux and energy cost for indoor wall and some for treadwall condition (Fig. 3) and, therefore, metabolic and hormonal response was “on average” similar between treadwall and indoor wall climbing in lower grade climbers as shown at Fig. 2.

There were no differences between climbing conditions for any subscale of CSAI or TFAI, nevertheless, advanced climbers showed significantly higher score for the regulatory dimension of perceived control (3.2 vs. 3.8, *P* = 0.03).

The video analysis showed no differences in any time-motion variables between treadwall and indoor wall climbing, however, advanced climbers demonstrated shorter mean time onto the hold and lower work/relief ratio (Table 2). This difference was significant for the non-dominant hand.

**Table 2:** Time motion analysis (mean ± *s*) of treadwall and indoor wall climbing in lower grade and advanced climbers.

Climbing ability	Treadwall		Indoor wall	
	Lower grade	Advanced	Lower grade	Advanced
Mean time on the hold NH (s)	*5.2 ± 0.3	*5.0 ± 0.3	*5.3 ± 0.5	*5.0 ± 0.4
Mean time on the hold DH (s)	5.2 ± 0.2	5.2 ± 0.2	5.3 ± 0.5	5.1 ± 0.4
Mean concurrent holding with DH and NH (s)	34.2 ± 16.9	51.1 ± 42.0	36.3 ± 16.1	40.6 ± 28.9
Work/relief ratio NH	*8.8 ± 5.0	*6.6 ± 2.6	*8.6 ± 3.6	*5.9 ± 1.1
Work/relief ratio DH	7.4 ± 1.8	7.5 ± 1.9	7.3 ± 1.3	7.1 ± 2.0

*NH* non-dominant hand, *DH* dominant hand.

*Statistical differences (*P* ≤ 0.05) between lower grade and advanced climbers.

## Discussion

The present study set out to compare participants’ psychophysiological response to two randomised, crossed-over ascents of identical routes on both a treadwall (climbing low to the ground) and an indoor wall (climbing in height) in a wide ability range of female climbers. The treadwall provides a wall climbing analogue that allows for control of climbing intensity low to the ground without additional security equipment. When these two methods were compared it was found that there was a systematically greater metabolic response for indoor wall climbing which was partially related to the catecholamine concentration increase that occurred during activity. Further, the advanced climbers showed smaller variation in metabolic and hormonal response between the two climbing conditions than lower grade climbers. No differences in time-motion parameters between the two climbing conditions were found.

Indoor climbing of the intensity used in the present study was found to have a substantial aerobic energy contribution with climbers achieving mean $\dot{V}$O_2_ values of 21–35 ml min^−1^ kg^−1^ which corresponds to previous findings^7,13,21,22^. The energy cost in climbing is mainly influenced by speed, wall inclination, climbers’ experience and overall difficulty of the route^23,24^ and climbing indoor route at an exhaustive intensity may increase $\dot{V}$O_2_ up to 44 ml min^−1^ kg^−1^^4^. Climbing on treadwall enables to move at higher speeds and peak $\dot{V}$O_2_ values of over 50 ml min^−1^ kg^−1^ have been reported, and treadwall climbing was proposed to be a good method to develop climbing specific aerobic fitness^10^. However, previous literature employing treadwall climbing has not been able to state if physiological responses are similar to the more complex indoor wall ascent, where psychological factors, such as fear from fall or height, may also affect the climber^10–12^. Our data show systematic elevated metabolic response when wall climbing, compared to the treadwall, although the potential fall was non-existent and the main difference was the height of the climb. The following text discusses potential mechanisms for the differences in metabolic response observed.

The use of anxiety inventories did not indicate any significant pre-ascent differences in any of the subscales between climbing conditions or ability groups. Advanced climbers scored higher in perceived control than lower grade climbers, which may be attributed to greater experience with climbing tasks. Climbing to height on an indoor wall induced greater catecholamine response to that close to the ground in lower grade, but not in advanced climbers and this was in part related to increased metabolic cost. The non-significantly greater catecholamine response in treadwall condition in advanced climbers may have resulted from the relative novelty of the task or be just coincidence. Figure 3 demonstrates very high intra-group catecholamine variability in lower grade climbers in both climbing conditions indicating that the stress responses of this group depended on many mechanisms not covered in the current study. For instance, some lower grade climbers exhibited greater metabolic and hormonal response in the treadwall than the indoor wall condition. As these climbers reported the lowest ability level from the whole group, it may be that the moving belt of the treadwall represented a more stressful condition than indoor wall climbing at height.

Another potential explanation for differences seen in the metabolic demand may be due to the amount of time spent in static positions. The length of static positions was included in the results as it may affect the metaboreflex and sympathetically mediated pressor response^25^. The video analysis did not show any differences between climbing conditions, however, advanced climbers demonstrated shorter time spent on holds and lower work/relief ratio. This is in agreement with previous findings^18^ and indicates longer inter-move recovery periods for the advanced climbers. This might have been related to the lower metabolic response seen in this group. Video analysis can only provide data on the time characteristics of applied load, while the intensity is not known. It has been shown that higher ability climbers apply less force on the holds which is associated with lower metabolic response^23^. However, as the applied force on the holds was not known in our study, its effect is only speculative.

Considering ability group differences, metabolic response (HR, $\dot{V}$_E_, and RER) were significantly elevated in lower grade than advanced climbers, which is in agreement with previous finding^17,22^. Lower metabolic cost in the advanced climbers may be accounted for by greater movement efficiency which is associated with several factors including inter- and intra-muscular coordination, route reading, fluency of movement, applied force distribution and/or also psychological stress^1,22,24,26^. In the current study, the differences in $\dot{V}$O_2_ between ability groups did not reach significance as hypothesised. This may be due to the intermediate climbers involved, it may be speculated that a greater difference in ability groups may have increased differences in metabolic and hormonal response in this study.

The main finding of the current study was that indoor wall climbing induced significantly greater metabolic response than a treadwall ascent, which was partially related to catecholamine increase during activity. The study advances our understanding of psychophysiological response in climbing as several biological, biomechanical and psychological markers were used. On the other hand, several limitations should be acknowledged. The study was undertaken with only female participants at one fixed climbing speed, which reduces the generalizability of the conclusions. While the speed used in the present study was at a typical pace^4^, it was not self-selected. The route was technically simple in vertical profile, it may be that technically more difficult, negatively or positively inclined walls would provide different results. Catecholamine and cortisol measures are notoriously variable and subject to experimental handling of sampling and processing. Moreover, asking participants about follicular phase provided us only “some” evidence. Nevertheless, measures of hormone concentration reproducibility would be very difficult in this experimental design as participants already provided several venous blood samples. Future research should explore different speeds and/or load application on holds to confirm our findings.

## Conclusion

Climbers are regularly exposed to physiological and to psychological stress when ascending routes. Treadwalls provide a physiological challenge where the risk of fall or fear from height is minimal and enables the analysis of physiological responses when climbing in a controlled setting. The present study demonstrates that treadwall climbing offers climbers, coaches and researchers with a wall climbing analogue, although they should be aware that it appears to induce a systematically lower metabolic response than indoor wall climbing, the exact mechanisms of which are not yet fully understood. Climbing to height on an indoor wall induced greater catecholamine response to that close to the ground in lower grade, but not in advanced climbers and this was in part related to increased metabolic cost. Time-motion characteristics and anxiety before the tasks appeared to be similar. Coaches should be aware of the concomitant psychophysiological demand of ascending to height, particularly in lower ability climbers.

## Data Availability

The datasets used and/or analysed during the current study are available from the corresponding author on reasonable request.
